# Mechanical Response and Microstructural Evolution Mechanisms of 2 vol.% TiB/Ti-55531 Composites During Isothermal Compression

**DOI:** 10.3390/ma19153276

**Published:** 2026-08-03

**Authors:** Nan Zong, Yongqiang Ye, Shaopeng Li, Yimin Zhuo, Hao Wang, Xue Zhang, Jianwen Le, Guangfa Huang, Jianwei Mao, Yuanfei Han, Weijie Lu

**Affiliations:** 1 The State Key Laboratory of Metal Matrix Composites, School of Materials Science and Engineering, Shanghai Jiao Tong University, Shanghai 200240, China; 018050210014@alumni.sjtu.edu.cn (N.Z.); yyqiang0522@126.com (Y.Y.); zhuoyimin24@sjtu.edu.cn (Y.Z.); wanghao2023@sjtu.edu.cn (H.W.); zxdut@sjtu.edu.cn (X.Z.); lejianwen@sjtu.edu.cn (J.L.); gavin@sjtu.edu.cn (G.H.); maojake@sjtu.edu.cn (J.M.); 2 AVIC Shenyang Aircraft Design & Research Institute, Shenyang 110035, China; lishaopeng601@163.com

**Keywords:** titanium matrix composites, metastable β titanium, hot deformation behavior, microstructural evolution

## Abstract

**Highlights:**

- Strain-compensated constitutive equations in α + β and β phase regions were established to capture flow behavior and predict flow stress of TiB/Ti-55531 composites.
- Three DRX mechanisms of β phase and two dynamic spheroidization mechanisms of α phase were identified. TiB plays a significant role in promoting β-DRX and tends to randomize crystallographic orientations of the β phase.
- A comprehensive microstructural evolution mechanism map was constructed and an optimized hot processing window was proposed by combining kinetic analysis and microstructural characterization.

**Abstract:**

Thermomechanical processing (TMP) is of critical importance for tailoring microstructures and properties of high-strength titanium alloys and their composites. In this study, mechanical response and microstructural evolution mechanisms of 2 vol.% TiB/Ti-5Al-5Mo-5V-3Cr-1Zr (Ti-55531) matrix composites during isothermal compression at varied deformation temperatures (785–925 °C) and strain rates (0.001–1 s^−1^) are comprehensively investigated by kinetic calculation and microstructural characterization. Strain-compensated constitutive equations in α + β and β phase regions were established. Results show that deformation temperature and strain rate influence flow behavior and microstructure through dynamic recovery (DRV) and dynamic recrystallization (DRX) of the β phase as well as dynamic spheroidization of the α phase. Crucially, three DRX mechanisms of β phase were identified, wherein TiB-induced β-DRX dominates, with α-assisted β-DRX and continuous dynamic recrystallization (CDRX) as secondary mechanisms. Dynamic spheroidization mechanisms of the α phase, including β-wedge penetration as well as α interaction and kinking, were elucidated. A comprehensive microstructural evolution mechanism map was constructed, and an optimized hot-processing window was proposed. Notably, the introduction of TiB significantly promotes β-DRX, which tends to randomize crystallographic orientations of the β phase, and enhances microstructural stability. This study provides theoretical complement and practical guidance for hot processing and microstructure control of metastable β titanium matrix composites.

## 1. Introduction

Ti-5Al-5Mo-5V-3Cr-1Zr (Ti-55531), as a metastable β titanium alloy, is widely used in the aerospace industry for load-bearing components such as aircraft landing gear and airframe fasteners owing to its ultra-high specific strength, good combination of strength and ductility, deep hardenability and high fatigue resistance, which can serve as a promising substitute for high-strength steels, achieving weight reduction up to 30% [1,2,3,4]. In order to further improve service performance to meet the increasingly stringent requirements of aerospace applications, titanium matrix composites that introduce ceramic reinforcements into titanium alloy matrix have attracted considerable research interest [5,6,7,8]. TiB is one of the ideal reinforcements owing to its high elastic modulus, similar density and Poisson’s ratio to those of titanium, small difference in thermal expansion, and no interface reaction with titanium [9,10].

The practical components made of titanium alloys need to undergo appropriate thermomechanical processing (TMP) and heat treatment to obtain specific microstructure, properties, shape, and size to meet the requirements of applications [11,12,13]. The microstructural evolution during TMP is rather complex due to the interplay of thermal and mechanical fields, which can induce dynamic recovery (DRV), dynamic recrystallization (DRX) of the β phase, and dynamic spheroidization of the α phase [14]. The deformation parameters such as deformation temperature and strain rate exert a significant influence on the resulting microstructure and dynamic mechanical response. Therefore, it is of great necessity to gain a thorough understanding of the hot deformation behavior and the associated microstructural evolution. A widely adopted approach in the study of hot deformation behavior involves establishing constitutive equations and hot processing maps through kinetic analysis of isothermal compression tests, which enables one to predict flow stress and optimize the processing parameters [15,16,17,18]. Combined with the observation and analysis of the retained microstructure, process optimization and microstructural control tend to be facilitated, benefitting the improvement of the service performance of the materials.

Up to now, extensive research has been conducted on the hot deformation behavior and microstructural evolution of Ti-55531 and other metastable β-titanium alloys. Zhou et al. [19] investigated the deformation mechanisms and constitutive considerations for Ti-55531 compressed at elevated temperatures; the deformation mechanism map was established based on the flow stress data, processing map, and grain morphology. Zhang et al. [20] examined the influence of strain rates on the hot deformation behavior and mechanisms of Ti-55531 during isothermal compression. The results show that discontinuous yielding behavior was observed at higher strain rates; the CDRX and DDRX of the β phase occurred at low strain rates, while DRV and CDRX occurred at high strain rates. Wu et al. [21] reported that the dynamic phase transformation behavior plays a main role in the softening at high strain rates of Ti-55531 during isothermal compression. Zhao et al. [22] conducted a comparative study on the hot deformation behavior and microstructural evolution of Ti-5553 fabricated by powder metallurgy and conventional melting, revealing the presence of continuous dynamic recrystallization (CDRX) at PM and discontinuous dynamic recrystallization (DDRX) at IM. In the subsequent related study of Zhao et al. [23], multiple α-spheroidization mechanisms were further identified. Ye et al. [24] systematically investigated the transition of deformation mechanisms in Ti1500G during hot compression and elucidated various dynamic recrystallization (DRX) mechanisms of the β matrix as well as the spheroidization of the α phase. Li et al. [25] reported the dynamic spheroidization mechanism of the α phase and the restoration mechanism of the β phase in Ti-5Al-2Sn-2Zr-4Mo-4Cr during isothermal compression. Another research of Li et al. [26] reported that the globular α grains assist in the DRX of the β phase during the deformation of the same alloy. Compared to titanium alloys, the hot working of titanium matrix composites is often rendered more complex by the incorporation of reinforcements.

Research on hot deformation behavior of titanium matrix composites has mainly concentrated on near-α and α + β titanium alloys [27,28,29,30,31], while studies on metastable β titanium matrix composites are relatively scarce. Yang et al. [32] studied the hot deformation behavior and microstructure evolution of (TiB + TiC)/Ti-3.5Al-5Mo-6V-3Cr-2Sn-0.5Fe via isothermal hot compression; the deformation activation energy and hot processing map were obtained. The results showed that the deformation mechanism in the β-phase region was associated with CDRX by lattice rotation, while it was associated with DRV and DRX when deformed in the(α + β) phase field. Yang et al. [33] investigated the microstructure evolution and texture evolution of 2 vol.% TiC/Ti-4Al-5Mo-5V-1Sn-2Zr composites during hot deformation. The results indicated that high deformation temperature and low strain rate are beneficial to promote DRX, and TiC plays a role in particle-stimulated nucleation during hot deformation. Anil et al. [34] examined the microstructural evolution and hot tensile deformation behavior of TiB/Ti-5Al-5V-5Mo-1Cr-1Fe composites and found that TiB led to significant grain refinement and changes in the morphology of α-phase from lamellar to globular.

Currently, published work is scarcely available regarding the hot deformation behavior and microstructural evolution of TiB/Ti-55531 composites, and related research is needed to fill this gap, which motivates this work. In this paper, 2 vol.% in situ synthesized TiB/Ti-55531 composites were prepared by vacuum consumable arc melting and then subjected to preliminary forging. Isothermal compression tests were performed at different temperatures and strain rates with 70% height reduction. A strain-compensated constitutive equation and processing maps were established through kinetic analysis. The microstructures under various deformation conditions were observed and analyzed to investigate the spheroidization mechanism of the α phase and the dynamic recrystallization mechanism of the β matrix. In addition, the influence of TiB reinforcement addition on the dynamic mechanical response and microstructural evolution was evaluated.

## 2. Materials and Methods

### 2.1. Materials

**Figure 1:**
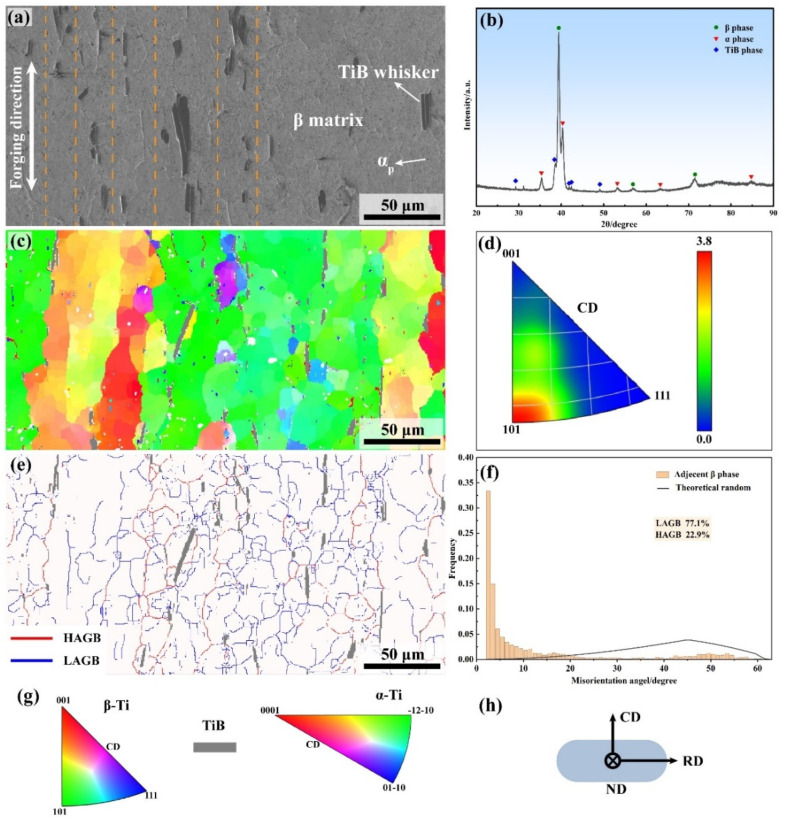
The initial microstructure and XRD pattern of TiB/Ti-55531 composites before compression: (**a**) SEM image, (**b**) XRD pattern, (**c**) grain orientation map, (**d**) inverse pole figure, (**e**) grain boundary map, (**f**) distribution of grain boundary misorientation map, (**g**) corresponding legend for (**c**,**e**), (**h**) processing direction indicator.

The materials used in this study are TiB/Ti-55531 matrix composites. The nominal composition of the matrix alloy is Ti-5Al-5Mo-5V-3Cr-1Zr, and the theoretical volume fraction of TiB reinforcement is 2%. First, the in situ synthesized cast ingot of titanium matrix composites (TMCs) was fabricated by vacuum consumable arc melting with sponge Ti (grade I, BaoTi group, Baoji, China), pure Al (Tianshan Aluminum Group, Shihezi, China), Ti-60Mo (50 wt.%, BaoTi group, China), Ti-85V (50 wt.%, BaoTi group, China), pure Cr (Beijing Xingrongyuan Technology, Beijing, China), sponge Zr (BaoTi group, China), and TiB_2_ powder (ATTL Advanced Materials, Beijing, China) as raw materials. The ingot was melted three times to improve the homogeneity of components, and TiB reinforcements were in situ synthesized via the reaction: TiB_2_ + Ti→2TiB. Next, the cast ingot was forged in the β phase and (α + β) phase region successively to obtain a square billet with a section size of 36 mm × 36 mm. The β-transus temperature is 855 °C ± 5 °C, measured by the metallographic method. The microstructure and XRD pattern of the forging billet are displayed in Figure 1. The SEM image (Figure 1a) shows that the original microstructure consists of the dominant β matrix phase, a tiny proportion of α phase, and TiB whiskers with the longitudinal axis parallel to the forging direction (FD). The compressive direction (CD) in subsequent isothermal compression tests was chosen to be parallel to FD. The XRD pattern in Figure 1b shows a consistent result with Figure 1a. Little information about the β matrix can be gained by SEM observation due to the corrosion resistance of the β phase. The EBSD results are displayed in Figure 1c–f. The grain orientation map captured along CD (Figure 1c) illustrates that the elongated β deformed structure shows a strong <101>//CD orientation and weak <100>//CD orientation, which is consistent with the result of the inverse pole figure (Figure 1d). The grain boundary map and the distribution of grain boundary misorientation (Figure 1e,f) demonstrate that a large number of low-angle grain boundaries (LAGB) exist in the elongated β deformed structure with a proportion of 77.1%, while the proportion of high-angle grain boundaries (HAGB) is only 22.9%. Here, the LAGB is defined as grain boundaries with a misorientation angle ranging from 2 to 15°, while HAGB refers to grain boundaries with a misorientation angle larger than 15°.

### 2.2. Isothermal Compression Tests

**Figure 2:**
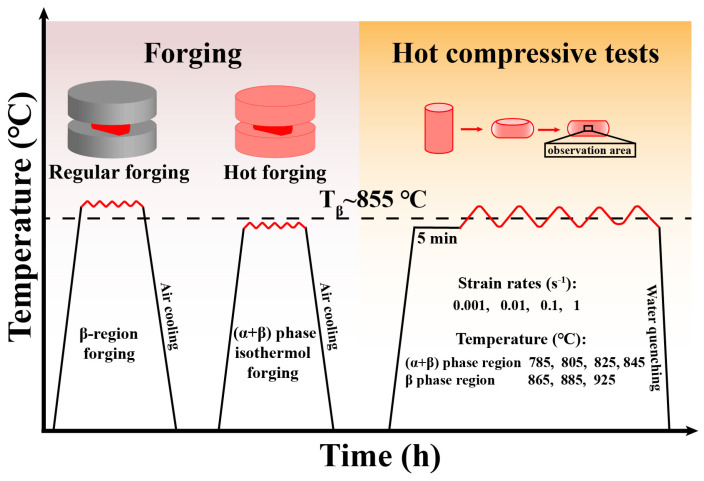
Schematic of the TMP route for TiB/Ti-55531 composites.

The isothermal compression tests were conducted on a Gleeble-3500 simulator in an argon atmosphere to protect samples from oxidation. The cylindrical samples with size of ϕ8 mm × 12 mm were prepared from the forging billet by wire electro-discharge machining with the CD parallel to FD and compressed at varied deformation temperatures (785 °C, 805 °C, 825 °C, 845 °C, 865 °C, 885 °C and 925 °C) and strain rates (0.001 s^−1^, 0.01 s^−1^, 0.1 s^−1^ and 1 s^−1^) with 70% height reduction. It is worth noting that the mechanical response and microstructural evolution may differ with varying compression directions. In the present study, this particular direction was chosen as the compression direction because the TiB whiskers are aligned parallel to it. Among all possible loading orientations, this direction imposes the most severe deformation conditions and exhibits the highest flow resistance, and thus represents the primary direction of concern for the hot deformation of the TiB/Ti-55531 composites. The K-type thermocouple was welded in the middle of the samples to measure the deformation temperature in real time. Tantalum sheets were placed between samples and indenters to reduce friction and improve deformation uniformity. First, samples were heated at a rate of 5 °C/s to the target deformation temperature and held for 300 s to ensure thermal equilibrium of the whole sample. Then, samples were compressed under the set deformation conditions. Finally, specimens were immediately quenched in water after deformation to retain the microstructure at high temperature. The TMP route is schematically illustrated in Figure 2. In this study, all specimens were machined to comparable dimensions with consistent surface quality, and the end faces were lubricated uniformly to minimize friction. Under these well-controlled conditions, a single specimen was tested per deformation condition, which is a widely adopted practice in hot compression studies [19,20,21,22,23,24,25].

### 2.3. Microstructure Characterization

A scanning electron microscope (SEM, NOVA NanoSEM 230, FEI, Hillsboro, OR, USA) equipped with electron backscatter diffraction (EBSD) and a transmission electron microscope (TEM, Talos F200S G2, Thermo Scientific, Waltham, MA, USA) were used to observe the microstructure of specimens. The central area of the longitudinal section of compressed samples was selected for microstructure observation, owing to the decreased influence of surface friction and temperature drop. The samples for SEM observation were prepared by conventional metallography techniques including grinding, mechanical polishing, and etching. The samples for EBSD observation were prepared by mechanical grinding, polishing, and vibration polishing successively. The final microstructures, including the sizes of β grains and α particles, varied with deformation temperature and strain rate. Consequently, different scan areas and step sizes were selected accordingly for EBSD characterization. For the specimens compressed in the α + β phase region, the EBSD scan area was 110 µm × 100 µm with a step size of 0.2–0.3 µm. For the specimens compressed in the β phase region, the scan parameters were set according to the strain rate: a scan area of 400 µm × 400 µm with a step size of 0.8 µm was used at 0.001 s^−1^, while a scan area of 250 µm × 250 µm with a step size of 0.4 µm was adopted for the other three strain rates. The EBSD data were processed by AztecCrystal software V2.1. The samples for TEM observation were first sliced from the middle of compressed specimens with a thickness of 0.8 mm parallel to the compressed axis, followed by mechanical grinding to 70 μm and punching into a disc with a diameter of 3 mm. Finally, the disc was ion-thinned until observable thin areas appeared.

## 3. Results

### 3.1. True Stress-Strain Curves

**Figure 3:**
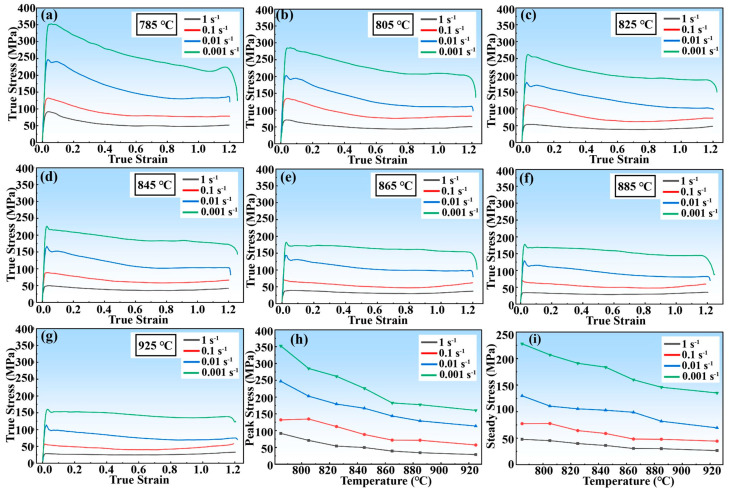
The true stress-strain curves, peak flow stress and steady-state flow stress of TiB/Ti-55531 composites at varied deformation conditions: (**a**) 785 °C, (**b**) 805 °C, (**c**) 825 °C, (**d**) 845 °C, (**e**) 865 °C, (**f**) 885 °C, (**g**) 925 °C, (**h**) peak flow stress at different temperatures and strain rates, (**i**) steady-state flow stress at different temperatures and strain rates.

The true stress-strain curves of TiB/Ti-55531 composites at varied deformation conditions are displayed in Figure 3a–g, Figure 3h,i show the variation of peak flow stress and steady-state flow stress at varied deformation conditions. In general, the true stress-strain curves at different compressive deformation parameters exhibit similar characteristics. The flow stress increases rapidly to the peak stress at the initial stage of deformation and then gradually decreases to a stable state. Thus, the compressive curves can be divided into three stages, namely the work hardening stage, flow softening stage, and steady flow stage. At the initial stage of deformation, the multiplication and entanglement of dislocations result in an increase in dislocation density and flow stress, which corresponds to the work hardening stage. With increasing strain, the occurrence of DRV, DRX of the β phase, and spheroidization of the α phase leads to a significant decrease in dislocation density. When the rate of dislocation annihilation exceeds the rate of dislocation induced by deformation, flow stress decreases accordingly, which corresponds to the flow softening stage. The multiplication and annihilation of dislocations reach equilibrium with further deformation, forming a steady flow stage. The flow stress increases with decreasing compressive temperature and increasing strain rate. The decrease in flow stress with increasing temperature can be attributed to several factors. First, higher temperatures enhance atomic kinetic energy and weaken atomic bonding, directly lowering the flow stress [28]. Second, more β phase with body-centered cubic (BCC) structure and less α phase with close-packed hexagonal (HCP) structure at higher temperatures reduce deformation resistance, due to the limited slip systems of α phase. Third, higher deformation temperatures promote DRV and DRX of the β phase, further reducing the flow stress. Regarding the increase in flow stress with increasing strain rate, there are two main reasons. First, the rapid multiplication of dislocations at higher strain rates leads to an increase in dislocation density, causing a higher flow stress. Second, insufficient time for DRV, DRX of the β phase, and spheroidization of the α phase results in a higher density of dislocations at higher strain rates, leading to a higher flow stress.

The discontinuous yielding phenomenon, which refers to an abrupt drop from the peak stress, occurs during isothermal compression at higher strain rates. The pinning of dislocations by a Cottrell atmosphere and depinning of dislocations from the Cottrell atmosphere are generally accepted explanations for discontinuous yielding [35]. Nevertheless, this theory fails to explain why discontinuous yielding typically occurs at higher strain rates. Therefore, the dynamic theory [36] that discontinuous yielding is related to the multiplication of movable dislocations was developed. According to dislocation theory, strain rate is proportional to mobile dislocation density and dislocation velocity, and dislocation velocity is positively related to flow stress. A sharp increase in mobile dislocation density at higher strain rates reduces dislocation velocity, resulting in an abrupt drop in flow stress.

### 3.2. Kinetic Analysis

Isothermal compression is a thermally activated process; the relationship between the flow stress σ (MPa), strain rate and deformation temperature T (K) is generally expressed in the form of the Arrhenius equation proposed by Sellars and Tegart [37]:

(1) $$\dot{\varepsilon }=\left\{\begin{array}{c} A_{1}\sigma^{n_{1}}exp\left(-\frac{Q}{RT}\right)\;(low\;stress\;level)\\ A_{2}exp\left(\beta \sigma \right)exp\left(-\frac{Q}{RT}\right)\;(high\;stress\;level)\\ A{\left[sinh\left(\alpha \sigma \right)\right]}^{n}exp\left(-\frac{Q}{RT}\right)\;(all\;stress\;levels) \end{array}\right.$$

where Q is deformation activation energy (kJ/mol), R is the molar gas constant (8.314 kJ/mol), $A_{1}$, $A_{2}$, A, $n_{1}$, n, β, α are material constants and $\alpha =\beta /n_{1}$. The hyperbolic sine function applicable to all stress levels is utilized to establish the constitutive equation in this study. Calculating the natural logarithm of both sides of Equation (1) yields Equation (2):

(2) $$ln\dot{\varepsilon }=\left\{\begin{array}{c} lnA_{1}+n_{1}ln\sigma -\frac{Q}{RT}\\ lnA_{2}+\beta \sigma -\frac{Q}{RT}\\ lnA+nln\left[sinh\left(\alpha \sigma \right)\right]-\frac{Q}{RT} \end{array}\right.$$

Equation (2) can be rearranged into Equation (3):

(3) $$\left\{\begin{array}{c} \alpha ={\left(\frac{\partial ln\dot{\varepsilon }}{\partial \sigma }\right)}_{T}\cdot {\left(\frac{\partial ln\sigma }{\partial ln\dot{\varepsilon }}\right)}_{T}\\ n={\left\{\frac{\partial ln\dot{\varepsilon }}{\partial ln\left[sinh\left(\alpha \sigma \right)\right]}\right\}}_{T}\\ Q=R{\left\{\frac{\partial ln\dot{\varepsilon }}{\partial ln\left[sinh\left(\alpha \sigma \right)\right]}\right\}}_{T}\cdot {\left\{\frac{\partial \ln[\sinh\left(\alpha \sigma )\right]}{\partial \left(T\right)}\right\}}_{\dot{\varepsilon }} \end{array}\right.$$

The values of α, n, and Q can be computed according to Equation (3). Introducing the Zener–Hollomon parameter (Z) into Equation (1) yields Equation (4):

(4) $$Z=\dot{\varepsilon }\;e{xp\left(\frac{Q}{RT}\right)}^{n}=\;A{\left[sinh\left(\alpha \sigma \right)\right]}^{n}$$

Calculating the natural logarithm of both sides of Equation (4) yields Equation (5):

(5) $$lnZ=\;ln\dot{\varepsilon }+\frac{Q}{RT}=lnA+nln\left[sinh\left(\alpha \sigma \right)\right]$$

**Figure 4:**
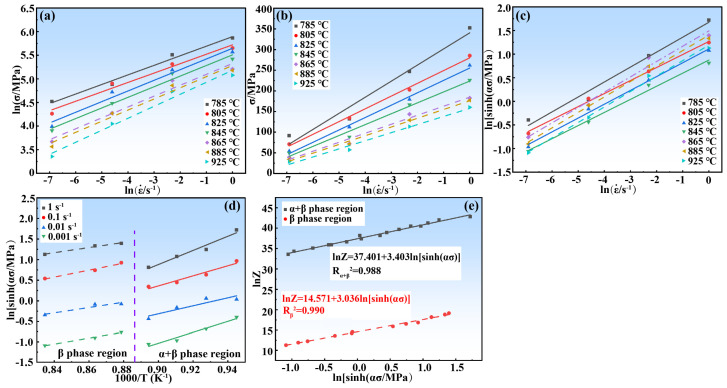
Linear relationships of (**a**) $ln\sigma -ln\dot{\varepsilon }$, (**b**) $\sigma -ln\dot{\varepsilon }$, (**c**) $ln\left[sinh\left(\alpha \sigma \right)\right]-ln\dot{\varepsilon }$, (**d**) $ln\left[sinh\left(\alpha \sigma \right)\right]-1000/T$, (**e**) $lnZ-ln\left[sinh\left(\alpha \sigma \right)\right]$ of TiB/Ti-55531 composites in the α+β phase region and the β phase region, respectively.

The value of lnA can be obtained by the interception of lnZ-$ln\left[sinh\left(\alpha \sigma \right)\right]$. The results of the aforementioned kinetic analysis are shown in Figure 4. The material constants and the corresponding constitutive equations in the α+β phase region and the β phase region are determined as:

(6) $$\dot{\varepsilon }=1.751\times 10^{16}{\left[\sinh\left(0.006867\sigma \right)\right]}^{3.452}\;exp\left(-\frac{376469}{RT}\right)$$

(7) $$\dot{\varepsilon }=2.129\times 10^{6}{\left[\sinh\left(0.01152\sigma \right)\right]}^{3.064}\;exp\left(-\frac{181358}{RT}\right)$$

It is worth mentioning that the calculated deformation activation energy in the α + β phase region is significantly higher than the self-diffusion activation energies of α-Ti and β-Ti [28]. This is due to the combined contribution of grain boundary migration of the β phase, spheroidization of the α phase, and obstruction of dislocation motion by TiB whiskers. These coordinated deformation processes require substantial energy to overcome the associated barriers, leading to an apparent activation energy well above the self-diffusion values. Furthermore, compared with the unreinforced Ti-55531 alloy [19,20,21], TiB/Ti-55531 exhibits a considerably higher activation energy. This increase is attributed to the introduction of TiB whiskers, which act as effective obstacles to dislocation motion and also contribute to load transfer strengthening. These combined effects significantly increase the deformation resistance and consequently raise the apparent activation energy [28].

**Figure 5:**
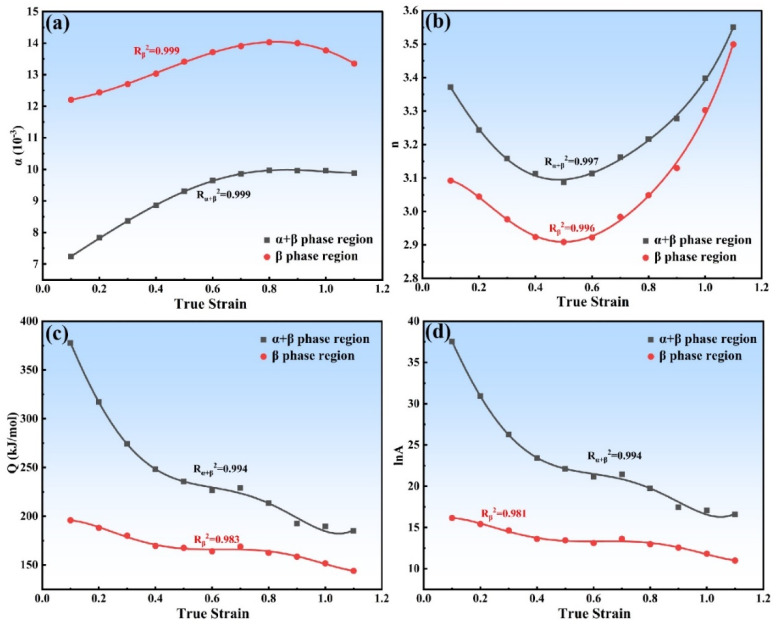
Variations of (**a**) α, (**b**) n, (**c**) Q, (**d**) ln(A) with true strain of TiB/Ti-55531 composites.

The aforementioned constitutive equations neglect the influence of strain on flow stress during compressive deformation. However, related research [38] indicates that material constants and deformation activation energy vary with strain. Strain-compensated constitutive equations are required to predict the flow stress more accurately. The material constants and deformation activation energy can be described as polynomial functions of strain. Related studies have shown that fifth-order or sixth-order polynomial fittings are commonly used [24,39]. In this study, a fifth-order polynomial fitting was adopted because it yields higher correlation coefficients (R^2^) compared with third-order, fourth-order, and sixth-order fittings, as demonstrated in Table S1. The fifth-order polynomial fitting utilized in this study is as follows:

(8) $$\left\{\begin{array}{c} \alpha =B_{0}+B_{1}\varepsilon +B_{2}\varepsilon^{2}+B_{3}\varepsilon^{3}+B_{4}\varepsilon^{4}+B_{5}\varepsilon^{5}\\ n=C_{0}+C_{1}\varepsilon +C_{2}\varepsilon^{2}+C_{3}\varepsilon^{3}+C_{4}\varepsilon^{4}+C_{5}\varepsilon^{5}\\ Q=D_{0}+D_{1}\varepsilon +D_{2}\varepsilon^{2}+D_{3}\varepsilon^{3}+D_{4}\varepsilon^{4}+D_{5}\varepsilon^{5}\\ lnA=E_{0}+E_{1}\varepsilon +E_{2}\varepsilon^{2}+E_{3}\varepsilon^{3}+E_{4}\varepsilon^{4}+E_{5}\varepsilon^{5} \end{array}\right.$$

where ε is strain, $B_{i}$, $C_{i}$, $D_{i}$ and $E_{i}$ (i = 0, 1, …, 5) are the coefficients of the polynomial function. The fitted lines are displayed in Figure 5, with the corresponding coefficients in the α + β phase region and the β phase region listed in Table 1 and Table 2, respectively.

It should be noted that the material constants and deformation activation energy in the constitutive equation represent the fitted average values over the entire phase region. To avoid confusion with the subsequent analysis of the activation energy variation across the selected temperature and strain rate ranges, the Q in both the constitutive equations and the strain-compensated constitutive models is hereafter referred to as the average deformation activation energy, while the Q presented in the activation energy contour maps is designated as the apparent deformation activation energy.

**Figure 6:**
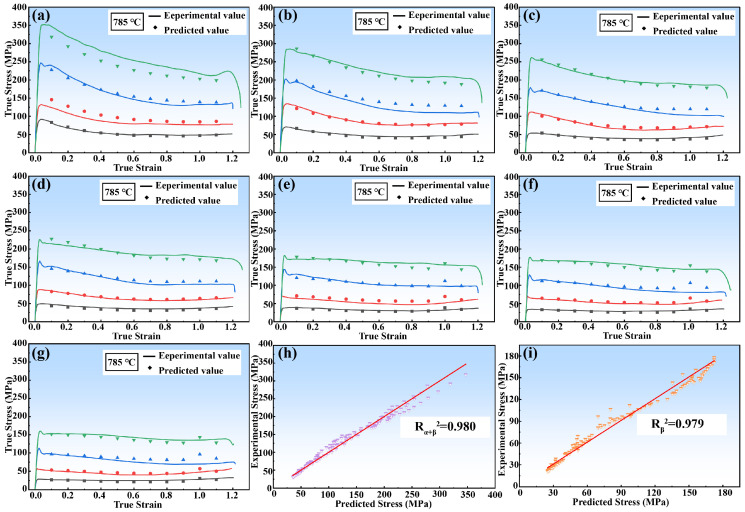
The comparison and correlation between experimental flow stresses and predicted flow stresses of TiB/Ti-55531 composites at varied deformation conditions: (**a**) 785°C, (**b**) 805°C, (**c**) 825°C, (**d**) 845°C, (**e**) 865°C, (**f**) 885°C, (**g**) 925°C, (**h**) correlation in the α + β phase region, (**i**) correlation in the β phase region.

The strain-compensated constitutive models of TiB/Ti-55531 composites are established at varied deformation conditions. The comparison and correlation between experimental flow stresses and predicted flow stresses are listed in Figure 6. The high coincidence degree between experimental flow stresses and predicted flow stresses shown in Figure 6 indicates the high accuracy of the established strain-compensated constitutive models. Specifically, the average absolute relative error (AARE) and correlation coefficient (R^2^) between the predicted and experimental flow stresses are 6.3% and 0.980 for the constitutive equation in the α + β phase region, and 7.1% and 0.979 for that in the β phase region, respectively.

**Figure 7:**
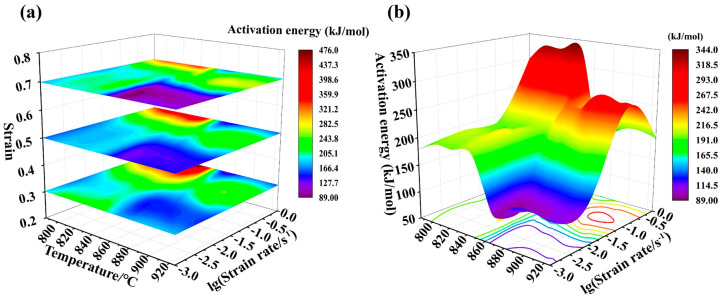
The apparent deformation activation energy of TiB/Ti-55531 composites: (**a**) 2D contour maps at true strains of 0.3, 0.5 and 0.7, (**b**) 3D contour map at true strain of 0.7.

The deformation activation energy is an indicator that reflects the difficulty of deformation during the thermal activation process, which is related to thermally activated mechanisms at the atomic scale [40]. The two-dimensional contour maps of Q at varied true strains (0.3, 0.5, and 0.7) and the corresponding three-dimensional contour map at a true strain of 0.7 are displayed in Figure 7. Q here stands for the apparent deformation activation energy. As shown in Figure 7, Q varies with deformation temperature, strain rate, and strain. The whole processing region is divided into four parts for better analysis of Q variation, namely the α + β phase region with high strain rates (I), α + β phase region with low strain rates (II), β phase region with high strain rates (III), and β phase region with low strain rates (IV). Generally, Q in the α + β phase region is evidently higher than that in the β phase region, indicating the higher deformation resistance in the α + β phase region. The existence of the α phase results in this phenomenon. On one hand, the α phase is more difficult to deform compared to the β phase owing to the limited slip systems. On the other hand, the pinning effect of the α phase on dislocations greatly hinders dislocation migration. Q tends to increase with increasing strain rate owing to the larger deformation resistance at higher strain rates. As strain increases, Q in regions I and IV tends to decrease, while Q in regions II and III decreases at the initial deformation stage and increases at the later deformation stage. This is likely related to DRV and DRX of the β phase during deformation. The occurrence of DRV and DRX at the initial deformation stage leads to flow softening, resulting in a decrease in deformation resistance and corresponding decrease in Q, while the growth of DRX grains at the later deformation stage results in an increase in Q. The dominant DRV and weaker DRX in region I and IV cause a monotonic decrease in Q as strain increases, while DRV and a higher degree of DRX in region II and III lead to a decrease in Q at first and gradually increase as deformation continues.

### 3.3. Hot Processing Map

The hot processing map is a powerful tool to evaluate the workability and optimize processing parameters, which is based on the dynamic materials model (DMM). According to DMM theory, the workpiece is treated as a power dissipater, and the whole deformation process is assumed to be a closed adiabatic system [41,42]. The total external power (P) consumed during the deformation process is dissipated into two parts:

(9) $$P=\sigma \dot{\varepsilon }=G+J=\int_{0}^{\dot{\varepsilon }}\sigma d\dot{\varepsilon }+\int_{0}^{\sigma }\dot{\varepsilon }d\sigma$$

where G represents the power dissipated by plastic deformation, and J is related to the power dissipated by the microstructural evolution.

The strain rate sensitivity coefficient (m), which determines the partitioning of G and J, is calculated by:

(10) $$m=\frac{\partial J}{\partial G}=\frac{\partial log\sigma }{\partial log\dot{\varepsilon }}$$

The power dissipation efficiency (η) is defined to quantify the fraction of power dissipated by microstructural evolution and can be expressed as:

(11) $$\eta =\frac{J}{J_{max}}=\frac{2m}{m+1}$$

The plot of η vs. deformation temperature (T) and strain rate $\dot{\left(\varepsilon \right)}$ constitutes a power dissipation map, illustrating the mechanical variation mechanisms at varied deformation conditions.

A criterion developed by Prasad for determining the flow instability is presented as [43]:

(12) $$\xi \left(\dot{\varepsilon }\right)=\frac{\partial log\left(\frac{m}{m+1}\right)}{\partial log\dot{\varepsilon }}+m<0$$

The plot of ξ with deformation temperature (T) and strain rate $\dot{\left(\varepsilon \right)}$ constitutes an instability map. When ξ is negative, flow instabilities such as flow localization and strain cracking are indicated to occur under the corresponding deformation conditions. The hot processing map is generally constructed by superimposing the instability map over the power dissipation map.

**Figure 8:**
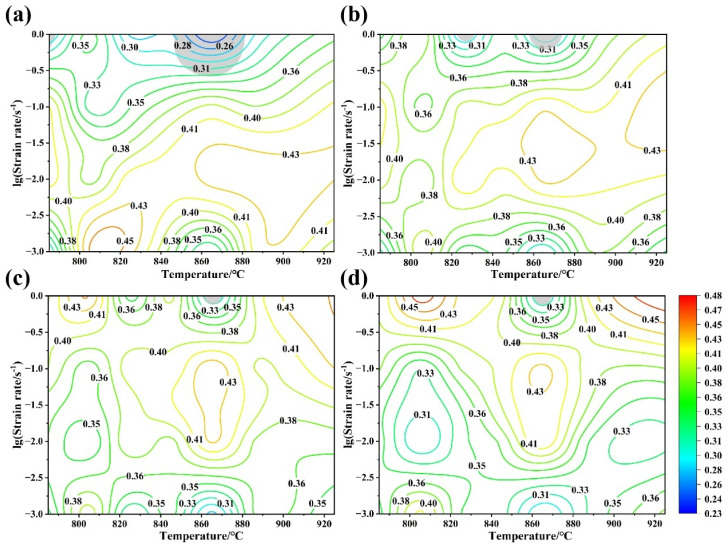
The hot processing maps of TiB/Ti-55531 composites at varied true strains: (**a**) 0.3, (**b**) 0.5, (**c**) 0.7, (**d**) 0.9.

Figure 8 presents the hot processing maps at varied true strains (0.3, 0.5, 0.7 and 0.9). Figure 8a shows that two instability regions indicated by grey occur at a true strain of 0.3, and they are located around 785 °C/0.7–1 s^−1^ and 850–880 °C/0.3–1 s^−1^. As true strain increases to 0.5 (Figure 8b), the instability regions become smaller; they are located at 825 °C/0.9–1 s^−1^ and 860–875 °C/0.6–1 s^−1^. As for true strains of 0.7 and 0.9 (Figure 8c,d), only a tiny instability region located around 865 °C/1 s^−1^ appears. Obviously, the instability region gradually shrinks with increasing strain at first and gradually becomes relatively stable. It can be attributed to the occurrence of DRV and DRX of the β phase during deformation, which results in the reduction or elimination of unstable microstructures such as flow localization. Overall, η is relatively high (>30%) in the selected processing window at varied strains, demonstrating a narrow instability region and a majority of safe processing intervals. The considerably high η at lower strain rates (>35%) throughout the entire deformation process indicates that it is a potential optimal processing window, which is consistent with the analysis of deformation activation energy. However, the determination of the optimal processing window requires further validation through microstructural characterization and analysis.

### 3.4. Microstructural Evolution

#### 3.4.1. Microstructure at Low Strain Rate in the α + β Phase Region

**Figure 9:**
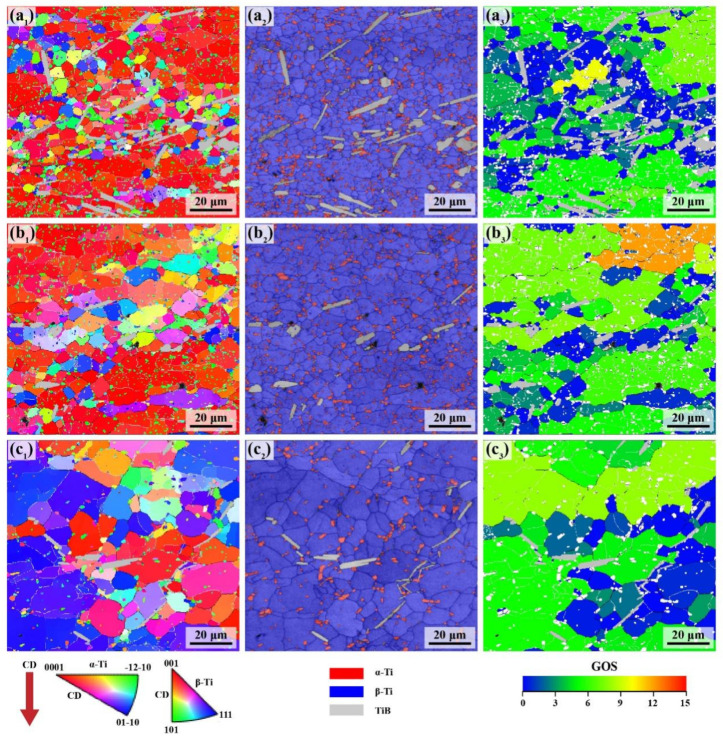
EBSD results of TiB/Ti-55531 composites compressed at low strain rate (0.001 s^−1^) and varying deformation temperatures in the α + β phase region: rows (**a_1_**–**a_3_**) 785 °C, (**b_1_**–**b_3_**) 805 °C, (**c_1_**–**c_3_**) 825 °C, columns (**a_1_**–**c_1_**) grain orientation maps, (**a_2_**–**c_2_**) phase distribution maps, (**a_3_**–**c_3_**) grain orientation spread (GOS) maps.

EBSD results of specimens deformed at low strain rate (0.001 s^−1^) and varying deformation temperatures (785 °C, 805 °C and 825 °C) in the α + β phase region are displayed in Figure 9, including grain orientation maps, phase distribution maps and grain orientation spread maps (GOS) of β phase. Grains displayed as different colors in orientation maps (Figure 9(a_1_–c_1_)) represent their different crystal orientations, and all grain orientation maps in subsequent figures are captured along CD. α phase, β phase, and TiB whiskers are displayed as different colors in phase distribution maps (Figure 9(a_2_–c_2_)). GOS maps (Figure 9(a_3_–c_3_)) show the degree of continuous change of orientation within a single grain. Grains with higher GOS generally undergo larger plastic deformation, while the newly formed DRX grains with little deformation are usually characterized by low GOS. Grains with GOS < 2° were identified as DRX grains according to related research [23,24]. The corresponding legends of EBSD maps are shown at the bottom of the figure.

As shown in Figure 9, the microstructure after compression presents significant differences from the initial microstructure before compression shown in Figure 1. TiB whiskers rotate gradually from the direction with the longitudinal axis parallel to CD to the direction with the longitudinal axis perpendicular to CD during isothermal compression. More equiaxed α phase particles are dispersed throughout the β matrix after compression, while an extremely small fraction of α phase existed in the initial microstructure. Equiaxed β grains with random orientations formed by DRX and β deformation substructures formed by DRV take the place of the stretched β deformation structures in the initial microstructure. For specimens deformed at 785 °C (Figure 9(a_1_–a_3_)), β-DRX grains with GOS < 2° are predominantly distributed near TiB whiskers with a proportion of about 32%, illustrating the facilitating effect of TiB whiskers on DRX. While DRV-induced β deformation substructures with higher GOS show a significant <001>//CD preferred orientation. Further increasing deformation temperature to 805 °C (Figure 9(b_1_–b_3_)) and 825 °C (Figure 9(c_1_–c_3_), the proportions of β-DRX grains become 25% and 29%, respectively. The fraction of TiB whiskers, deformation temperature, and the resulting variation of α phase collectively influence DRX, leading to this result. First, the observed region of the specimen deformed at 785 °C contains a higher fraction of TiB whiskers (6.7 vol.%) compared with those deformed at 805 °C (2.2 vol.%) and 825 °C (2.3 vol.%), which is conducive to the occurrence of DRX. Second, higher deformation temperature can facilitate the nucleation and growth of DRX by lowering the critical strain and accelerating grain boundary migration [44,45]. Third, α phase particles dispersed in the β matrix can facilitate DRX [24]. The fraction of the α phase for the specimen deformed at 785 °C is 9%, with an average size of 1.14 μm. As deformation temperature increases to 805 °C and 825 °C, the fraction of α phase decreases to 7.9% and 4.8%, while the corresponding average size increases to 1.21 μm and 1.4 μm. A higher fraction of finer α phase particles dispersed in the β matrix is conducive to DRX for specimens deformed at lower deformation temperatures. The combined effects of the aforementioned three factors result in varied proportions of β-DRX grains. For specimens deformed at 785 °C, the promoting effect of TiB on DRX, along with the promoting effect arising from the high fraction and small size of α-phase particles, significantly outweighs the suppressing effect of low deformation temperature, resulting in the highest degree of DRX. For specimens deformed at 805 °C and 825 °C, the promoting effect of TiB on DRX is comparable. The promoting effect of increasing deformation temperature on DRX outweighs the suppressing effect caused by the reduction fraction and increased size of α-phase particles. Therefore, the specimen deformed at 825 °C exhibits a higher degree of DRX than the specimen deformed at 805 °C.

#### 3.4.2. Microstructure at Medium Strain Rates in the α + β Phase Region

**Figure 10:**
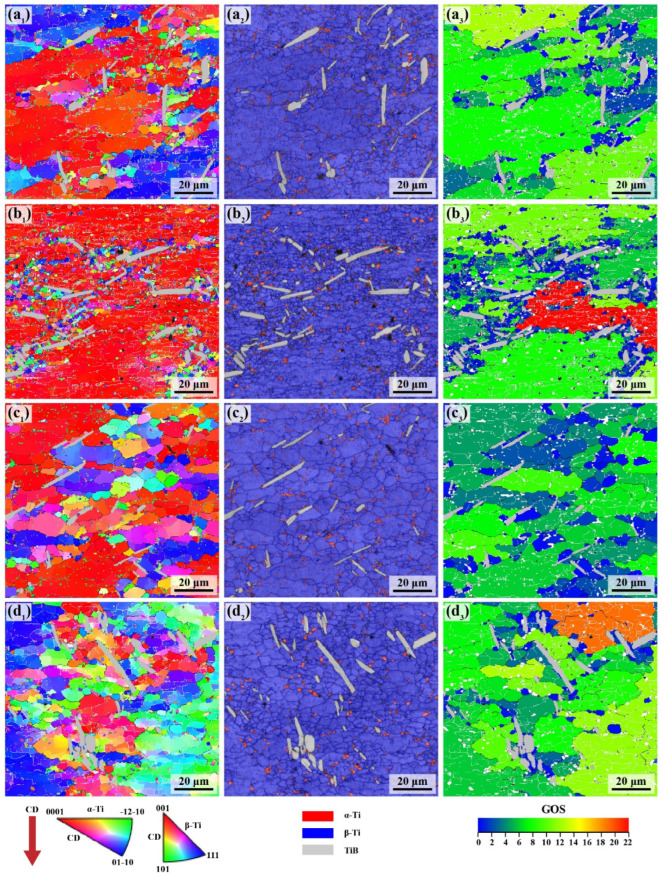
EBSD results of TiB/Ti-55531 composites compressed at medium strain rates (0.01 s^−1^ and 0.1 s^−1^) and varying deformation temperatures in the α + β phase region: rows (**a_1_**–**a_3_**) 785 °C–0.01 s^−1^, (**b_1_**–**b_3_**) 785 °C–0.1 s^−1^, (**c_1_**–**c_3_**) 825 °C–0.01 s^−1^, (**d_1_**–**d_3_**) 825 °C–0.1 s^−1^, columns (**a_1_**–**d_1_**) grain orientation maps, (**a_2_**–**d_2_**) phase distribution maps, (**a_3_**–**d_3_**) GOS maps.

Figure 10 presents the EBSD results of specimens deformed at medium strain rates (0.01 s^−1^ and 0.1 s^−1^) and varying deformation temperatures (785 °C and 825 °C) in the α + β phase region. As shown in Figure 10(a_1_–a_3_,c_1_–c_3_), the fractions of β-DRX grains with GOS < 2° for specimen deformed at 785 °C–0.01 s^−1^ and 825 °C–0.01 s^−1^ are 10% and 17%, respectively. Similar TiB volume fractions (approximately 3%) are measured in the two specimens. The promoting effect of increasing deformation temperature on DRX plays a dominant role, while the suppressing effect caused by the reduced fraction and enlarged size of α phase particles induced by increasing deformation temperature plays a secondary role. Therefore, the degree of DRX for the specimen deformed at 825 °C–0.01 s^−1^ is higher than that for the specimen deformed at 785°C–0.01 s^−1^. As for specimen deformed at 785 °C–0.1 s^−1^ (Figure 10(b_1_–b_3_)) and 825°C–0.1 s^−1^ (Figure 10(d_1_–d_3_)), the significant promoting effect induced by more TiB whiskers (5.6 vol.%) in the observed region of the specimen deformed at 785 °C–0.1 s^−1^ on DRX, along with the promoting effect from α phase particles (high fraction and small size), overcomes the suppressing effect of low deformation temperature, resulting in a significantly higher degree of DRX for specimen deformed at 785 °C–0.1 s^−1^ than 825°C–0.1 s^−1^.

Compared to Figure 9 in Section 3.4.1, the fraction of β-DRX grains decreases significantly as strain rate increases from low to medium. The effect of strain rate on DRX is multifaceted. First, DRV, serving as the dominant softening mechanism during deformation at higher strain rates, consumes most of the deformation energy, resulting in the inhibition of DRX [46]. Second, an increase in strain rate raises the critical strain for DRX initiation [44], resulting in the inhibition of DRX. Third, the higher density of dislocations during deformation at higher strain rates, which hinders grain boundary migration [45], coupled with the shorter deformation time, prevents DRX grains from growing sufficiently, causing the suppression of DRX. Last, the significant reduction of α-phase particles during deformation at higher strain rates leads to the suppression of DRX. The combined influence of the aforementioned factors leads to a reduction in the fraction of β-DRX grains as the strain rate increases.

Notably, as strain rate increases from 0.01 s^−1^ to 0.1 s^−1^, the fraction of α phase particles decreases, whereas the average size of α phase particles increases. As shown in Figure 10(a_1_–a_3_,b_1_–b_3_), the fraction and size of α phase particles change from 4.2% and 1.03 μm to 3.6% and 1.31 μm, respectively. A similar trend can also be observed by comparing Figure 10(c_1_–c_3_,d_1_–d_3_). The fraction and size of α phase particles change from 4.1% and 1.07 μm to 2.8% and 1.32 μm, respectively. Two factors account for the reduction in the fraction of α phase. First, deformation at higher strain rates increases the driving force for the dynamic α-β phase transformation, leading to a reduction in the fraction of α phase [47]. Second, more deformation heat generated during deformation at higher strain rates promotes further dissolution of α phase, causing a reduction in the fraction of α phase. The increase in the average size of α phase particles can be attributed to the insufficient spheroidization of α phase particles resulting from the limited deformation time at higher strain rates.

In summary, the fraction of TiB whiskers, deformation temperature, strain rate, and the resulting variation of α phase (fraction and size) collectively influence DRX of β phase during isothermal compression, leading to the variation in the degree of DRX.

#### 3.4.3. Microstructure at High Strain Rate in the α + β Phase Region

**Figure 11:**
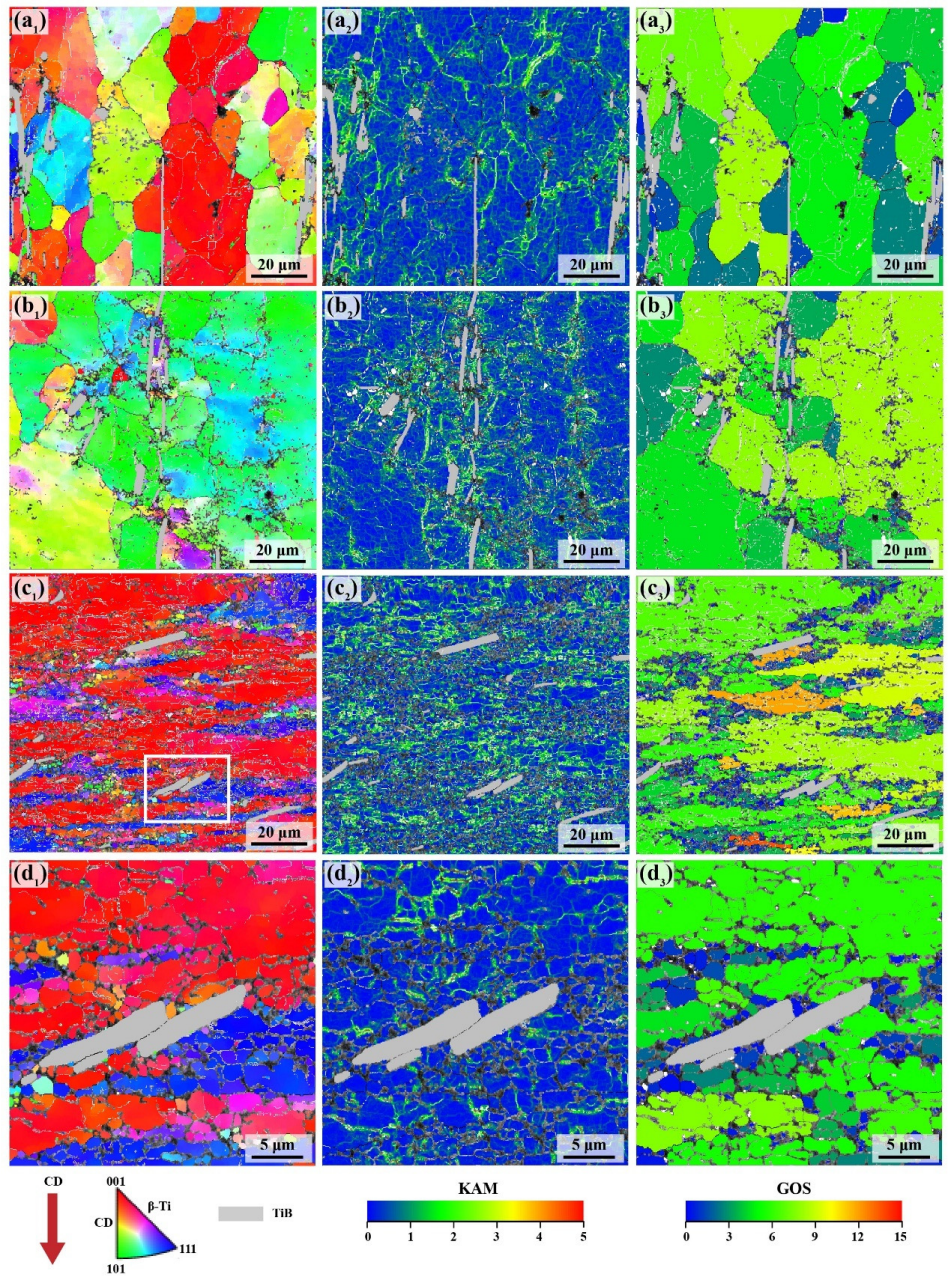
EBSD results of TiB/Ti-55531 composites compressed at high strain rate (1 s^−1^) and varying deformation temperatures in the α + β phase region: rows (**a_1_**–**a_3_**) 785 °C, (**b_1_**–**b_3_**) 805 °C, (**c_1_**–**c_3_**) 825 °C, (**d_1_**–**d_3_**) enlarged images of the white-framed region in (**c_1_**), columns (**a_1_**–**d_1_**) grain orientation maps, (**a_2_**–**d_2_**) kernel average misorientation (KAM) maps, (**a_3_**–**d_3_**) GOS maps.

EBSD results of specimens deformed at high strain rates (1 s^−1^) and varying deformation temperatures (785 °C, 805 °C and 825 °C) in the α + β phase region are displayed in Figure 11. Except for grain orientation maps and GOS maps mentioned above, kernel average misorientation (KAM) maps are used to represent the dislocation density distribution throughout the observed area. Higher KAM means higher dislocation density. As shown in Figure 11(a_1_–a_3_,b_1_–b_3_), the microstructure of specimens deformed at 785 °C and 805 °C exhibits similar characteristics to the original microstructure. TiB whiskers maintain the original arrangement with the direction of longitude axis parallel to CD, showing no noticeable rotation. The stretched β deformation structures exhibit pronounced orientations, and a quite small fraction of α phase exists. The internal dislocation density is relatively high, particularly at some specific locations such as the vicinity of TiB whiskers and grain boundaries. Some regions cannot even be indexed due to the excessive distortion. DRX rarely occurs, and the stretched β deformation structures with relatively low GOS illustrate the insufficient DRV. Apart from a high density of dislocations at specific locations, the observed regions overall show relatively minor plastic deformation and remain similar to the initial microstructure. However, flow localization can be observed in both specimens, and representative morphological images from the specimen compressed at 785 °C–1 s^−1^ are shown in Figure S1. Figure S1 reveals that TiB whiskers are extensively broken within the flow localization regions. This is attributed to the more severe stress concentration during deformation at relatively low temperature and high strain rate, particularly in the vicinity of TiB whiskers. The occurrence of flow localization and TiB fracture indicates that this processing condition is unsuitable, which is partially consistent with the instability domain predicted in Figure 8 in Section 3.3. It should be noted that the final processing window should be established by integrating kinetic calculations with detailed microstructural observations, as a processing map based solely on the instability criterion serves only as a reference. As deformation temperature increases to 825 °C (Figure 11(c_1_–c_3_)), the typical microstructure of compressive deformation induced by DRV and DRX of the β phase occurs, and TiB whiskers rotate in tandem with the rotation of β matrix grains. For better observation and visualization, EBSD results obtained with a finer step size at larger magnification of the white-frame region in Figure 11(c_1_) are presented in Figure 11(d_1_–d_3_). As shown in Figure 11(d_1_–d_3_), β-DRX grains occur in the vicinity of TiB whiskers, the α phase particles, and grain boundaries. A large number of sub-grains are generated inside β deformation substructures induced by DRV. Increasing deformation temperature enhances atomic mobility, inducing intensified dislocation movement and rearrangement. As a result, DRX and DRV of the β phase are facilitated compared to specimens deformed at lower deformation temperatures.

#### 3.4.4. Microstructure in the β Phase Region

**Figure 12:**
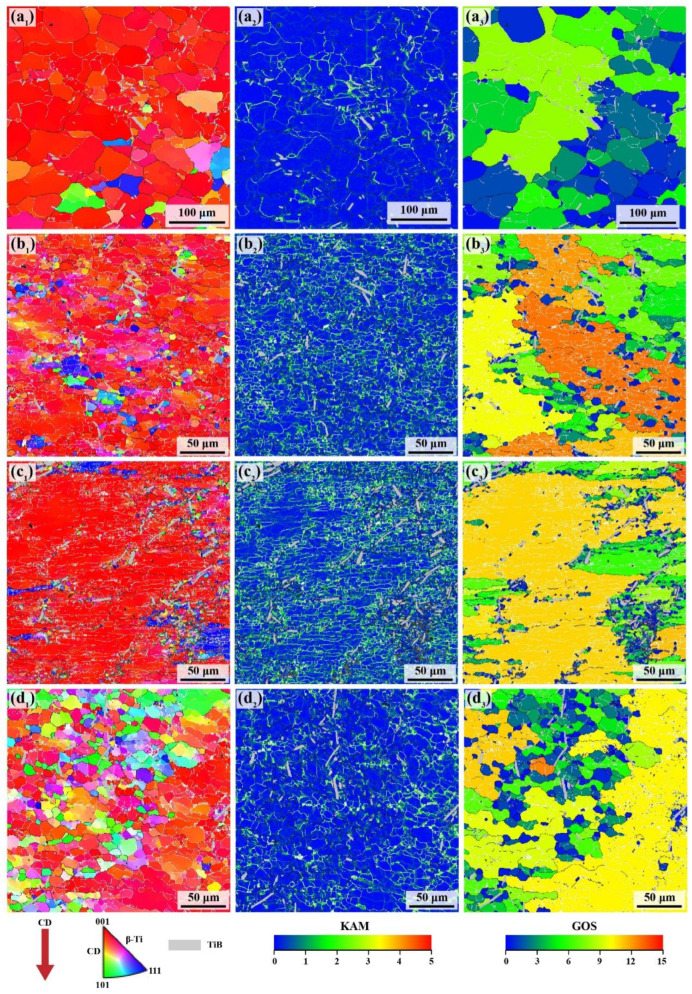
EBSD results of TiB/Ti-55531 composites compressed in the β phase region: rows (**a_1_**–**a_3_**) 865 °C–0.001 s^−1^, (**b_1_**–**b_3_**) 865 °C–0.1 s^−1^, (**c_1_**–**c_3_**) 865 °C–1 s^−1^, (**d_1_**–**d_3_**) 885 °C–0.1 s^−1^, columns (**a_1_**–**d_1_**) grain orientation maps, (**a_2_**–**d_2_**) KAM maps, (**a_3_**–**d_3_**) GOS maps.

Figure 12 displays the EBSD results of specimens deformed in the β phase region at different deformation conditions. As shown in Figure 12(a_1_–c_1_), β grains with relatively random orientations formed by DRX and β deformation substructures induced by DRV occur in the microstructure of specimens deformed at 865 °C and varying strain rates (0.001 s^−1^, 0.1 s^−1^ and 1 s^−1^). β deformation substructures show a significant <001>//CD preferred orientation. Corresponding KAM maps (Figure 12(a_2_–c_2_)) demonstrate that the dislocation density increases with strain rate, owing to the increase in dislocation multiplication rate and the suppression of DRV resulting from limited deformation time. Corresponding GOS maps (Figure 12(a_3_–c_3_)) illustrate that the fraction of β-DRX grains decreases with strain rate. The competition between DRV and DRX, along with the limited deformation time during deformation at higher strain rates, results in this phenomenon. The opposite variation in the size of β-DRX grains arises from the limited space induced by a higher nucleation rate and limited time for grain growth during deformation at higher strain rates.

As deformation temperature increases to 885 °C at 0.1 s^−1^ (Figure 12(d_1_–d_3_)), the dislocation density decreases and the fraction of β-DRX grains increases compared to Figure 12(b_1_–b_3_). Higher deformation temperature can promote dislocation slipping, climbing, and rearrangement, thereby facilitating DRX of the β phase. The intensification of DRV and DRX induced by higher deformation temperature leads to the reduction of dislocation density. Notably, DRV and DRX compete with each other at high strain rates, whereas they act synergistically at low or medium strain rates.

In general, higher deformation temperatures and low strain rates are conducive to DRV and DRX of the β phase, resulting in a more homogeneous plastic deformation and a more stable microstructure. However, excessively high temperatures and low strain rates can induce post-DRV and grain coarsening, which can adversely affect the performance of the material.

## 4. Discussion

As mentioned in Section 3, DRX of the β phase and dynamic spheroidization of the α phase occur during isothermal compression. β-DRX grains can occur at different locations, such as the vicinity of TiB whiskers, the vicinity of α phase particles, grain boundaries, and grain interiors. The corresponding DRX mechanisms are different. The DRX mechanisms of the β phase and the dynamic spheroidization mechanisms of α phase will be discussed in the following section in detail.

### 4.1. TiB-Induced β-DRX Mechanism

**Figure 13:**
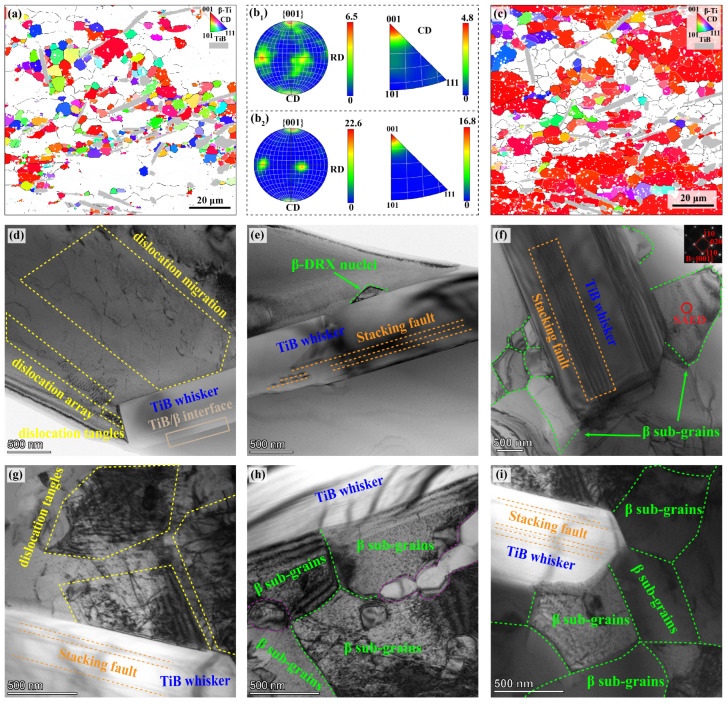
TiB-induced β-DRX behavior of TiB/Ti-55531 composites: (**a**) grain orientation map of β grains with GOS < 2° at 785°C–0.001 s^−1^, (**b_1_**,**b_2_**) corresponding pole figures and inverse pole figures of β grains with GOS < 2° and GOS > 2°, (**c**) grain orientation map of β grains with GOS > 2° at 785 °C–0.001 s^−1^, (**d**–**f**) TEM bright field images of 825 °C–0.01 s^−1^, (**g**–**i**) TEM bright field images of 825 °C–1 s^−1^.

EBSD and TEM results demonstrating the TiB-induced DRX behavior of TiB/Ti-55531 composites are displayed in Figure 13. Grain orientation map of β grains with GOS < 2° at 785 °C–0.001 s^−1^ (Figure 13a) shows that the majority of β-DRX grains with relatively random orientations are located in the vicinity of TiB whiskers. Corresponding {001} pole figure and inverse pole figure along CD are presented in Figure 13(b_1_). {001} pole figure illustrates that β-DRX grains show a weak {001}<100> texture with the maximum Intensities of 6.5 times the random texture, and a weak <001>//CD fiber texture with the maximum intensities of 4.8 can be observed from the inverse pole figure along CD. The results from these three figures are consistent, indicating that the β-DRX grains possess relatively random orientations and exhibit a weak texture. GRain orientation map of β grains with GOS > 2° at 785 °C–0.001 s^−1^ presented in Figure 13c demonstrates that β deformation substructures show a significant <001>//CD preferred orientation. Corresponding {001} pole figures and inverse pole figure along CD are displayed in Figure 13(b_2_), illustrating that β deformation substructures show a strong {001}<100> texture with the maximum intensities of 22.6 and a weak <001>//CD fiber texture with the maximum intensities of 16.8. The consistent results seen from these three figures show that β deformation substructures possess non-random orientations and exhibit a strong texture. The maximum pole density in the TiB-adjacent DRX regions is considerably lower than that in the substructure regions relatively distant from the TiB whiskers, which are induced by DRV. It is widely recognized that DRV is the predominant restoration mechanism in the Ti-55531 alloy during hot deformation, regardless of whether deformation occurs in the α + β or β phase regions [48], which typically results in deformation substructures with strong preferred orientations. In addition, a similar observation has been reported in a study on isothermally forged TiB/Ti-55531 composites, where TiB was found to effectively promote DRX and consequently lead to grain orientation randomization of β phase [49]. Based on the above observations and discussion, it is indicated that the introduction of TiB promotes β-DRX, and the resultant β-DRX grains tend to randomize the crystallographic orientations to some extent.

Figure 13d–f are TEM bright field images of specimen compressed at 825 °C–0.01 s^−1^. As shown in Figure 13d–f, the interface between TiB whiskers and β matrix shows excellent bonding. The parallel straight lines observed inside TiB whiskers are stacking faults, which frequently appear in TiB with B27 structure [50]. As a hard ceramic phase, TiB is extremely difficult to deform and can act as an obstacle to dislocation migration, causing dislocation tangles and accumulation in the vicinity of TiB, thereby forming a gradient deformation zone. The aforementioned phenomenon can be observed from Figure 13d. The gradient deformation region generated near TiB whiskers can act as a favorable site for the nucleation of β-DRX grains. As deformation progresses to a certain extent, β-DRX nuclei and β-DRX sub-grains occur in the vicinity of TiB whiskers, as shown in Figure 13e,f. Some HAGBs of β-DRX grains can be generated directly owing to the large misorientation between the newly formed β-DRX nuclei and the adjacent deformed β grains, while others are converted from LAGBs by the progressive rotation of adjacent β-DRX sub-grains. An increasing number of β-DRX sub-grains and grains are generated in the vicinity of TiB whiskers as the strain increases, and the newly formed β-DRX grains can grow through grain boundary migration or sub-grain coalescence during the subsequent deformation. The TEM results of the specimen deformed at higher strain rate (825 °C–1 s^−1^) displayed in Figure 13g–i reveal similar TiB-induced DRX behavior. As the strain rate increases, dislocation tangles and accumulations become more intense, and more β-DRX sub-grains and grains tend to nucleate in the vicinity of TiB whiskers, but the competition of adjacent grains and the limited deformation time constrains the growth of the newly formed β-DRX grains.

### 4.2. CDRX Mechanism of β Phase

**Figure 14:**
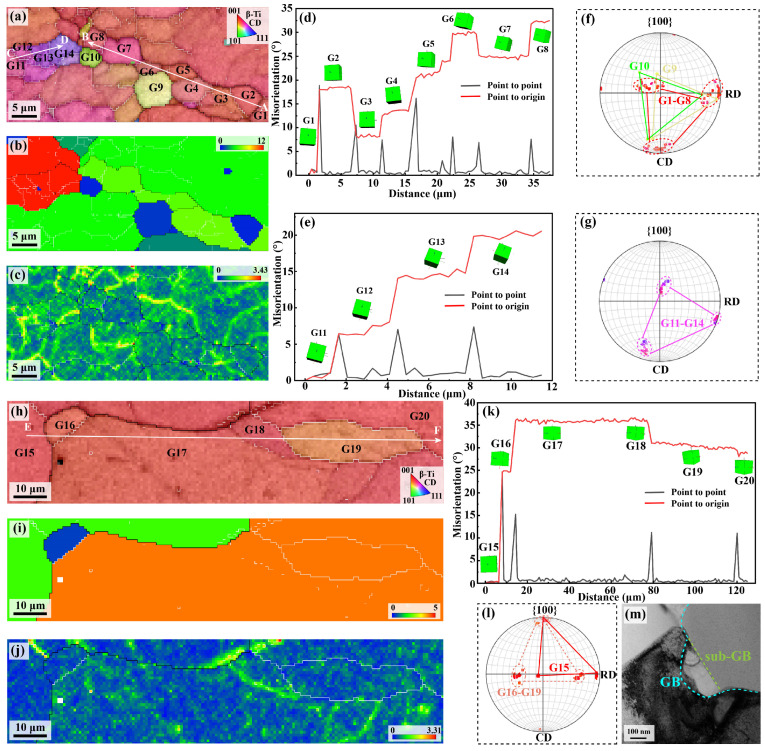
CDRX behavior of TiB/Ti-55531 composites: (**a**) grain orientation map of 865 °C–0.1 s^−1^, (**b**) corresponding GOS map, (**c**) corresponding KAM map, (**d**) variations of misorientation angles along AB, (**e**) variations of misorientation angles along CD, (**f**) pole figure of G1−G10, (**g**) pole figure of G11–G14, (**h**) grain orientation map of 865 °C–0.001 s^−1^, (**i**) corresponding GOS map, (**j**) corresponding KAM map, (**k**) variations of misorientation angles along EF, (**l**) pole figure of G15–G20, (**m**) corresponding bright field image.

The EBSD and TEM results demonstrating the CDRX behavior of TiB/Ti-55531 composites are presented in Figure 14. Figure 14a–g exhibit the CDRX behavior within the deformed β grains, while Figure 14h–m show the CDRX behavior at grain boundaries of the deformed β grains. The grain orientation map GOS map and KAM map shown in Figure 14a–c are utilized to distinguish β-DRX grains and β deformation substructures of the specimen deformed at 865 °C–0.1 s^−1^. The selected grains G1–G10 and G11–G14 are β-DRX grains or sub-grains at the formative stage. The cumulative misorientation (point to origin) and local misorientation (point to point) along the vectors AB and CD labelled in Figure 14a are calculated to reflect the orientation changes of G1–G8 and G11–G14. The results are shown in Figure 14d,e, respectively. Corresponding {100} pole figures of G1–G10 and G11–G14 are displayed in Figure 14f,g, respectively. Figure 14d shows that the misorientations within G1–G8 are quite small while the misorientations between G1–G8 possess varied misorientations between each other. As shown in Figure 14f, the poles of G1–G10 can be superimposed through low-angle rotations, suggesting a globally similar orientation among the grains. The results of Figure 14d–f demonstrate that the progressive rotation of sub-grains increases their mutual misorientations, and the β-DRX grains gradually generate such as G9 and G10. This represents a typical CDRX process, wherein DRX grains are generated from the progressive rotation of sub-grains [51]. The results of Figure 14e–g exhibit a similar process: the sub-grains G11–G14 generated by DRV gradually increase misorientations by progressive rotation, suggesting that DRV provides prior microstructural preparation for DRX and that they work synergistically to facilitate microstructural evolution during plastic deformation.

It is worth noting that CDRX generally occurs within deformed grains, whereas DDRX typically takes place at grain boundaries, in which new grains nucleate via grain boundary bulging and subsequently grow through grain boundary migration [51]. As can be seen from Figure 14h–j, DRX at grain boundaries is observed in the specimen deformed at 865 °C–0.001 s^−1^. The calculated results of cumulative misorientation and local misorientation along the vectors EF labelled in Figure 14h are displayed in Figure 14k, showing that the misorientations inside G16–G19 are quite small while the local misorientation between G16 and G17 reaches about 15°. Corresponding {100} pole figure of G15–G20 shown in Figure 14l indicates that G16–G19 exhibit a similar orientation while G15 exhibits a quite different orientation. The results of Figure 14k,l illustrate that the β-DRX grain at grain boundary (G16) is generated by the progressive rotation of sub-grains, which represents a CDRX process. The tension resulting from dislocations accumulated at grain boundaries leads to grain boundary bowing. Although it appears similar characteristics to the grain boundary bulging nucleation observed in DDRX, the underlying mechanisms are distinctly different. Corresponding TEM bright field image is captured and displayed in Figure 14m, which mutually validates the EBSD results.

### 4.3. α-Assisted β-DRX Mechanism

**Figure 15:**
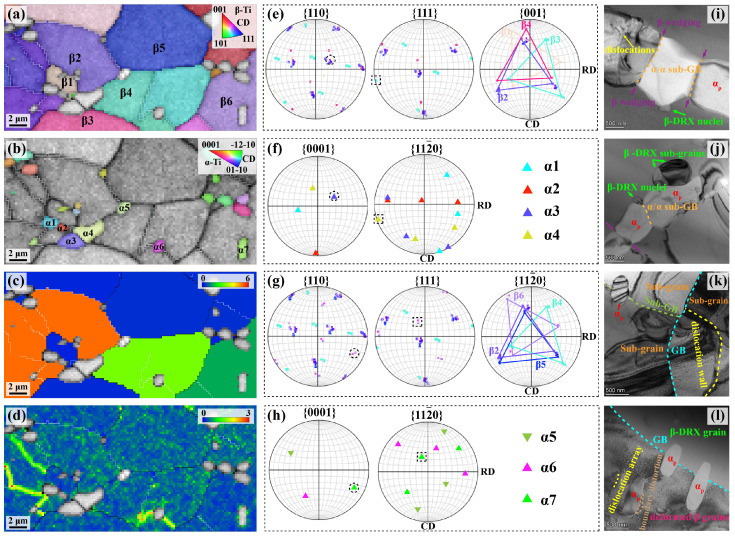
α-assisted DRX behavior of TiB/Ti-55531 composites: (**a**) grain orientation map of β phase of 825 °C–0.001 s^−1^, (**b**) corresponding grain orientation map of α phase, (**c**) corresponding GOS map of β grains, (**d**) corresponding KAM map of β grains, (**e**) pole figure of β1–β4, (**f**) pole figure of α1–α4, (**g**) pole figure of β2 and β4–β6, (**h**) pole figure of α5–α7, (**i**–**l**) corresponding bright field images.

The EBSD and TEM results illustrating the α-assisted DRX behavior of TiB/Ti-55531 composites are displayed in Figure 15. Grain orientation map of the β phase, grain orientation map of the α phase, GOS map of the β phase, and KAM map of the β phase are used to distinguish the β-DRX grains in the vicinity of α phase particles of the specimen deformed at 825 °C–0.001 s^−1^ (Figure 15a–d), showing that β1 is a DRX grain generated around α1–α4. {110}, {111} and {100} pole figures of β1 and its adjacent grains β2-β4 are presented in Figure 15e. As shown in Figure 15e, β1 exhibits a quite different orientation from β2–β4, which illustrates that β1 nucleates directly with the assistance of the surrounding α particles, rather than by progressive rotation of the surrounding β sub-grains. Generally, α and β phases obey the Burgers orientation relationship (BOR) during phase transformation under near-equilibrium conditions in titanium alloys, given by {0001}_α_//{110}_β_ and <11$\bar{2}$0>_α_//<111>_β_ [24]. {0001} and {1$\bar{2}$10} pole figures of α1–α4 are exhibited in Figure 15f. As shown in Figure 15e,f, no BOR is observed between α1, α2 and the adjacent grains β1–β4. α3 exhibits a partial BOR with β2, characterized by {0001}_α_//{110}_β_. α4 exhibits a partial BOR with β4, characterized by <11$\bar{2}$0>_α_//<111>_β_. The results illustrate that the high distortion energy arising from high-density dislocations accumulated at the α/β interface partially or completely destroys the BOR between the α and β phases during isothermal deformation. Based on the above analysis, the mechanism of α-assisted DRX of the β phase can be determined. The difference in deformation capability between α and β phases renders the α/β interface a favorable site for dislocation accumulation. The stored distortion energy associated with high-density dislocations enables the α/β interface to serve as a preferential nucleation site for DRX, thereby promoting the occurrence of β-DRX grains. Corresponding TEM bright-field images exhibiting α-assisted DRX of β phase are captured at a specimen deformed at 825 °C–0.01 s^−1^ and displayed in Figure 15i,j. It can be seen from Figure 15i,j that β-DRX nuclei and sub-grains occur at the α/β interface, which can mutually validate and supplement the aforementioned EBSD results.

As observed in Figure 15a–d, in addition to inducing the direct nucleation of β-DRX, α phase particles are also associated with the formation of β-DRX sub-grains and the growth of β-DRX grains. Three α grains located at grain boundaries (α5), sub-grain boundaries (α6), and the grain interior (α7) were selected for further analysis. {110}, {111} and {100} pole figures of the adjacent β grains (β2 and β4–β6) are displayed in Figure 15g. {0001} and {1$\bar{2}$10} pole figures of α5–α7 are presented in Figure 15h. As shown in Figure 15g,h, α7 located at the grain interior exhibits BOR with its adjacent grain β7, whereas α5 located at the grain boundaries and α6 located at sub-grain boundaries completely deviate from the BOR with their adjacent β grains. It can be inferred from the above results that the pinning effect of α phase particles on dislocations during plastic deformation promotes the formation of dislocation walls and sub-grain boundaries, thereby facilitating the CDRX process, whereas α phase particles located at grain boundaries inhibit the growth of β-DRX grains [52]. Corresponding TEM bright-field images presenting this phenomenon are captured at the specimen deformed at 825 °C–0.01 s^−1^ and displayed in Figure 15k,l, which can mutually validate and supplement the aforementioned EBSD results.

It is worth mentioning that the discussion on the α-assisted DRX mechanism is based on several selected grains in the analyzed region. In the full scanned area of this specimen, multiple instances of α-assisted β-DRX are observable by combining the BOR map and GOS map, as presented in Figure S2. Statistical analysis of the BOR relationship between α and the β matrix across the entire scanned region reveals that approximately 64% of the α particles deviate from BOR with β. These α particles are predominantly located at grain boundaries and sub-grain boundaries, where they promote CDRX of β while suppressing the growth of β-DRX grains. In contrast, about 24% of α particles maintain BOR with the β matrix and are mainly distributed within the grain interiors.

**Figure 16:**
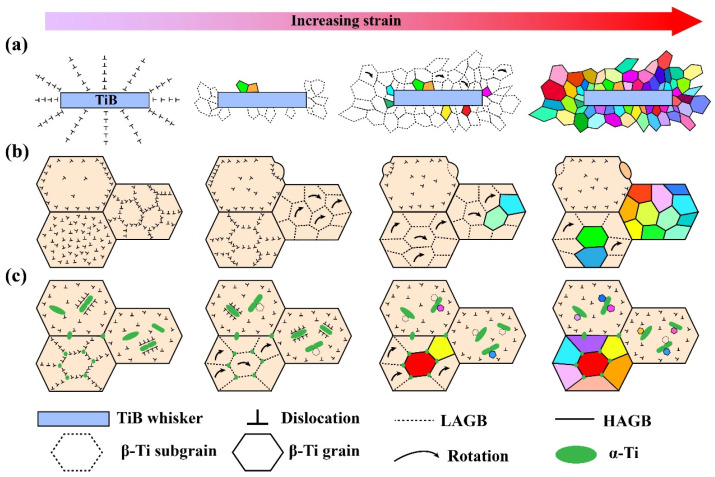
Schematic diagrams illustrating the DRX mechanisms of TiB/Ti-55531 composites: (**a**) TiB-induced DRX mechanism, (**b**) CDRX mechanism, (**c**) α-assisted DRX mechanism.

Based on the above analysis and discussion, schematic diagrams showing these three DRX mechanisms of the β phase during isothermal compression of TiB/Ti-55531 composites are displayed in Figure 16. Figure 16a exhibits the TiB-induced DRX mechanism. Dislocations accumulate in the vicinity of TiB whiskers, forming a gradient deformation zone. β-DRX nuclei and sub-grans tend to nucleate in the zone with high-density dislocations near TiB whiskers. As strain increases, β-DRX sub-grains undergo progressive rotation, transforming LAGBs into HAGBs to generate β-DRX grains. Concurrently, an increasing number of β-DRX sub-grains and grains are generated. Figure 16b illustrates the CDRX mechanism, including intragranular CDRX and grain boundary CDRX. In intragranular CDRX, dislocation slipping, dislocation climbing, and dislocation rearrangement take place during deformation, leading to the generation of β-DRX sub-grains. These sub-grains undergo progressive rotation, which increases the misorientation, thereby resulting in β-DRX grains. As for grain boundary DRX, dislocations accumulated at grain boundaries cause grain boundary bowing under tension. The bowed grain boundaries, along with LAGBs formed during DRV, constitute sub-grains. These sub-grains undergo progressive rotation to generate β-DRX grains during subsequent deformation. Figure 16c presents the α-assisted DRX mechanism. On the one hand, dislocation accumulation at α/β interface provides a preferential nucleation site for β-DRX sub-grains or grains, and these sub-grains transform into β-DRX grains during subsequent deformation. On the other hand, α phase particles promote the formation of sub-grains during the CDRX process, but inhibit the growth of DRX grains.

### 4.4. Dynamic Spheroidization Mechanism of α Phase

**Figure 17:**
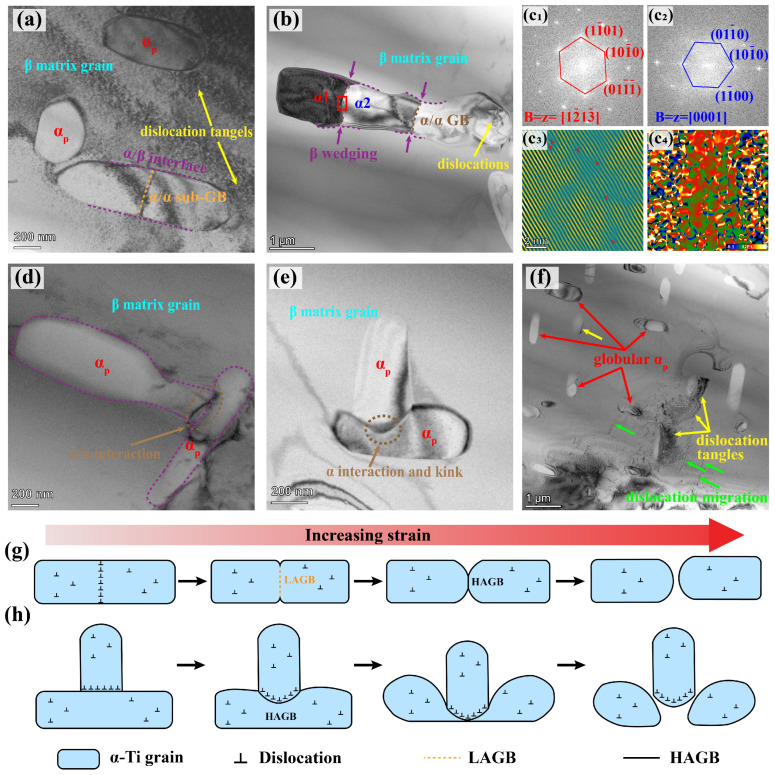
Dynamic spheroidization behavior of α phase in TiB/Ti-55531 composites: (**a**,**b**) bright field images of 825 °C–0.01 s^−1^, (**c_1_**,**c_2_**) corresponding fast Fourier transform (FFT) patterns of α grains marked with red frame in (**b**), (**c_3_**) corresponding inverse fast Fourier transformation (IFFT) image of the interior of α grain, (**c_4_**) corresponding geometric phase analysis (GPA) image of α grains marked with red frame in (**b**), (**d**–**f**) bright field images of 825 °C–0.01 s^−1^, (**g**,**h**) corresponding schematic of teo dynamic spheroidization mechanisms of α phase, respectively.

According to the results of Section 3, dynamic spheroidization of the α phase takes place during isothermal deformation of TiB/Ti-55531 composites. TEM results captured in specimens deformed at 825 °C–0.01 s^−1^ illustrating dynamic spheroidization mechanisms of the α phase are displayed in Figure 17a–f. As can be observed from Figure 17a, DRV within the α phase results in dislocation rearrangement, thereby forming α/α sub-grain boundaries. When α/α sub-grain boundaries form an angle of approximately 90° with the adjacent α/β phase boundaries, the interfacial configuration becomes energetically unfavorable. To lower the interfacial energy, β phase penetrates along α/α sub-grain boundaries, resulting in the formation of β wedges [23]. As deformation proceeds, the penetration of β wedges gradually deepens, while α/α sub-grain boundaries progressively transform from LAGBs into HAGBs. The corresponding bright-field image is presented in Figure 17b. Further analysis of the α/α interface marked by the red square in Figure 17b is displayed in Figure 17(c_1_–c_4_). The fast Fourier transform (FFT) images of two α grains on either side of the α/α interface (α1 and α2) are presented in Figure 17(c_1_) and Figure 17(c_2_), respectively. As shown in Figure 17(c_1_,c_2_), the zone axes of α1 and α2 are [1$\bar{2}$1$\bar{3}$] and [0001], respectively. The significant misorientation (>15°) between the zone axes of α1 and α2 indicates that the α/α interface has already transformed into HAGB. The inverse fast Fourier transformation (IFFT) image of α1 shown in Figure 17(c_3_) suggests that a small number of randomly distributed dislocations are present within α grain. The result of geometric phase analysis (GPA) analysis displayed in Figure 17(c_4_) demonstrates that α/α interface exhibits a significant strain gradient, promoting penetration of β wedges and subsequent separation and spheroidization of α phase.

Figure 17d,e show another dynamic spheroidization mechanism of the α phase via α lamellae interaction and kinking. As shown in Figure 17d, an α/α interface is formed when two α lamellae come into contact. Dislocations tend to accumulate at the α/α interface during deformation. The high-energy state of the interfacial configuration, along with the accumulation of high-density dislocations, leads to the bowing of the α/α interface and the preferential penetration of the β phase. The interaction and kinking of α lamellae can be observed during this process, as shown in Figure 17e. As the penetration of the β phase intensifies, the connecting region of the kinking α lamella is completely separated, forming isolated α fragments, which eventually spheroidize under the driving force of interfacial energy. Dynamic spheroidization of the α phase is achieved through the above two mechanisms, as shown in Figure 17f. Corresponding schematic diagrams of these two mechanisms are displayed in Figure 17g and Figure 17h, respectively.

### 4.5. Microstructural Evolution Mechanism Map

**Figure 18:**
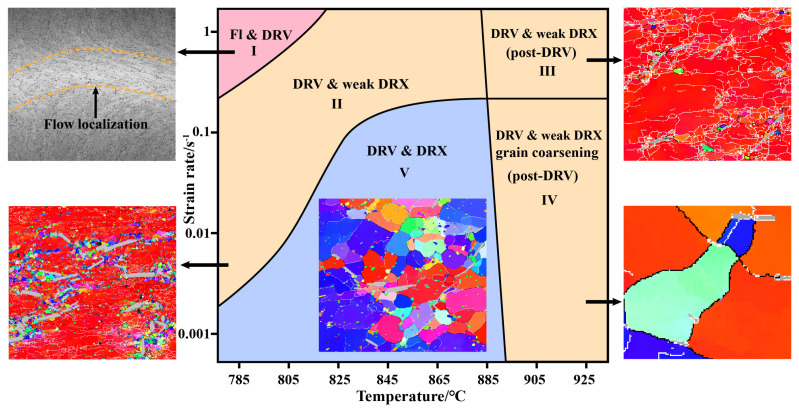
Schematic map demonstrating the dominant microstructural evolution mechanisms of TiB/Ti-55531 composites during isothermal compression at varied temperatures and strain rates.

Based on the analysis and discussion of kinetic calculation and microstructural characterization, a comprehensive schematic map demonstrating the dominant microstructural evolution mechanisms of TiB/Ti-55531 composites during isothermal compression at varied deformation temperatures and strain rates is presented in Figure 18. In region I (low deformation temperatures and high strain rates), DRV serves as the dominant deformation mechanism, while DRX rarely occurs. The resulting microstructure consists mainly of elongated β deformation structures, which are characteristic of the original microstructure. Flow localization (FL) potentially occurs at specific spots due to the local stress concentration induced by the accumulation of high-density dislocations. This is not an ideal processing window, which should be avoided in practical processing. Region II can be attained by appropriately increasing the deformation temperature or decreasing the strain rate of region I. In region II, DRV remains dominant, but a certain degree of DRX occurs. The resulting microstructure is composed of β deformation substructure induced by DRV along with a small fraction of DRX grains. Further increasing the deformation temperature from region II leads to region III. In region III the dominant mechanisms remain consistent with those in region II, namely DRV and weak DRX. Notably, post-DRV tends to occur in certain grains. Region IV can be reached upon a further reduction in strain rate from region III. In region IV, in addition to DRV, weak DRX and post-DRV as described in region III. Grain coarsening additionally takes place. Although these three processing windows (II, III and IV) rarely exhibit rheological instabilities such as flow localization, they are characterized by relatively uneven distribution of plastic deformation and inhomogeneous microstructures. Therefore, they are secondary processing windows that require careful selection. As for the remaining region V, it covers low temperatures and low strain rates regime as well as medium temperature and medium-to-low strain rates regime. In region V, DRV is sufficiently developed and the proportion of DRX is relatively high. The overall plastic deformation is relatively uniformly distributed and the microstructure is correspondingly homogeneous. Region V is recommended as the optimal processing window due to the high processing stability.

## 5. Conclusions

In the present study, the hot deformation behavior and microstructural evolution of 2 vol.% TiB/Ti-55531 matrix composites during isothermal compression at varied deformation temperatures (785–925 °C) and strain rates (0.001–1 s^−1^) with 70% reduction are comprehensively investigated by kinetic calculation and microstructural characterization, revealing mechanical response and microstructural evolution mechanisms. The main conclusions are as follows:

(1) The true stress-strain curves at varied deformation conditions exhibit similar morphologies, including work hardening stage, flow softening stage, and steady flow stage. Discontinuous yielding occurs at higher strain rates. The flow stress decreases with deformation temperature and increases with strain rate.
(2) Strain-compensated constitutive equations in α + β and β phase regions are established that can describe the flow behavior and accurately predict the flow stress.
(3) DRV and DRX of β phase, dynamic spheroidization of α phase, and rotation of TiB whiskers occur during isothermal compression. Deformation temperature and strain rate regulate the microstructure by affecting these processes.
(4) Three DRX mechanisms are identified. TiB-induced β-DRX is dominant, while the α-assisted β-DRX and CDRX are secondary. The introduction of TiB significantly promotes DRX of the β phase and tends to randomize crystallographic orientations of the β phase. Two dynamic spheroidization mechanisms of the phase are identified. One involves wedge penetration of the β phase, the other involves the interaction and kinking of α phase.
(5) Based on deformation activation energy analysis, hot processing maps and microstructural characterization, a comprehensive map of microstructural evolution mechanism with varying deformation temperature and strain rate is established, and an optimized processing window is determined.

## Figures and Tables

**Table 1:** The polynomial fitting results of α, n, Q and lnA in the α + β phase region.

α		n		Q		lnA	
B0	0.00666	C0	3.51073	D0	450.54600	E0	45.50058
B1	0.00581	C1	−1.37057	D1	−748.34383	E1	−81.78797
B2	−0.00002	C2	−0.95332	D2	−56.83077	E2	−6.82453
B3	0.00036	C3	7.58012	D3	3086.20517	E3	341.94423
B4	−0.00698	C4	−8.67564	D4	−4245.35232	E4	−470.51301
B5	0.00409	C5	3.30017	D5	1698.45160	E5	188.24824

**Table 2:** The polynomial fitting results of α, n, Q and lnA in the β phase region.

α		n		Q		lnA	
B0	0.0121	C0	3.06801	D0	188.9778	E0	15.41465
B1	0.00048	C1	0.89637	D1	185.35474	E1	19.31406
B2	0.00627	C2	−8.09795	D2	−1478.25889	E2	−150.69937
B3	−0.00212	C3	17.83382	D3	3310.62689	E3	338.18375
B4	−0.00495	C4	−15.56131	D4	−3033.72346	E4	−310.89216
B5	0.002	C5	5.14997	D5	978.09226	E5	100.4359

## Data Availability

The original contributions presented in this study are included in the article. Further inquiries can be directed to the corresponding authors.

## Supplementary Materials

The following supporting information can be downloaded at: https://www.mdpi.com/article/10.3390/ma19153276/s1, Figure S1. Morphologies of TiB/Ti-55531 composites compressed at 785 °C–1 s^−1^ (a) low magnification, (b) high magnification; Figure S2. α-assisted DRX behavior of TiB/Ti-55531 composites compressed at 825 °C–0.001 s^−1^ (a) BOR map, (b) GOS map; Table S1 Correlation coefficients (R^2^) of polynomial fitting with different orders for α, n, Q and lnA in the α+β phase region and β phase region.
