# Supplementary material for: Mechanical Response and Microstructural Evolution Mechanisms of 2 vol.% TiB/Ti-55531 Composites During Isothermal Compression
Source: Materials (Basel). 2026 Aug 3;19(15):3276. doi: 10.3390/ma19153276 (PMC13467489; doi:10.3390/ma19153276)
Supplement: Supplementary file 1 [file materials-19-03276-s001.zip › materials-4404991-supplementary.pdf]

Mechanical Response and Microstructural Evolution Mechanisms of 2 vol.% TiB/Ti-55531 Composites During Isothermal Compression

Nan Zong [1], Yongqiang Ye [1], Shaopeng Li [2], Yimin Zhuo [1], Hao Wang [1], Xue Zhang [1], Jianwen Le [1], Guangfa Huang [1], Jianwei Mao [1], Yuanfei Han [1,*] and Weijie Lu [1,*]

[1] The State Key Laboratory of Metal Matrix Composites, School of Materials Science and Engineering, Shanghai Jiao Tong University, Shanghai 200240, China; 018050210014@alumni.sjtu.edu.cn (N.Z.); yyqiang0522@126.com (Y.Y.); zhuoyimin24@sjtu.edu.cn (Y.Z.); wanghao2023@sjtu.edu.cn (H.W.); zxdut@sjtu.edu.cn (X.Z.); lejianwen@sjtu.edu.cn (J.L.); gavin@sjtu.edu.cn (G.H.); maojake@sjtu.edu.cn (J.M.)

[2] AVIC Shenyang Aircraft Design & Research Institute, Shenyang 110035, China; lishaopeng601@163.com

* Correspondence: hyuf1@sjtu.edu.cn (Y.H.); luweijie@sjtu.edu.cn (W.L.)

1. Comparison of correlation coefficients (R^2) for polynomial fittings with different orders for α, n, Q and lnA in the α+β phase region and β phase region

Table S1 Correlation coefficients (R^2) of polynomial fitting with different orders for α, n, Q and lnA in the α+β phase region and β phase region

		α	n	Q	lnA
Third-order	$R^2_{\alpha+\beta}$	0.997	0.991	0.980	0.980
	R^2_{β}	0.999	0.994	0.970	0.967
Fourth-order	$R^2_{\alpha+\beta}$	0.999	0.995	0.991	0.991
	R^2_{β}	0.999	0.993	0.968	0.965
Fifth-order	$R^2_{\alpha+\beta}$	0.999	0.997	0.994	0.994
	R^2_{β}	0.999	0.996	0.983	0.981
Sixth-order	$R^2_{\alpha+\beta}$	0.999	0.996	0.993	0.993
	R^2_{β}	0.999	0.996	0.978	0.976

2. Flow localization observed in TiB/Ti-55531 composites compressed at 785 ℃-1 s^{-1}

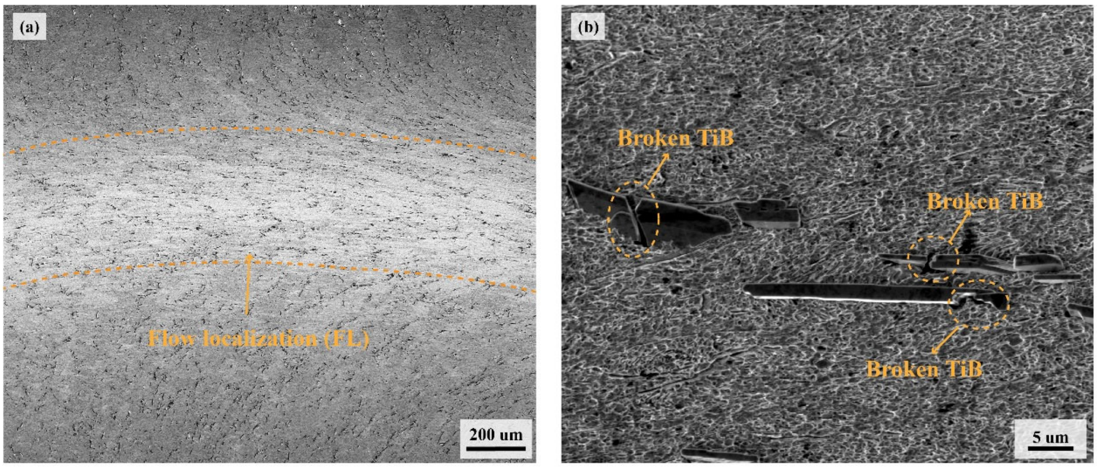

Figure S1. Morphologies of TiB/Ti-55531 composites compressed at 785 ℃-1 s^{-1} (a) low magnification, (b) high magnification

3. EBSD results illustrating α-assisted DRX behavior of TiB/Ti-55531 composites

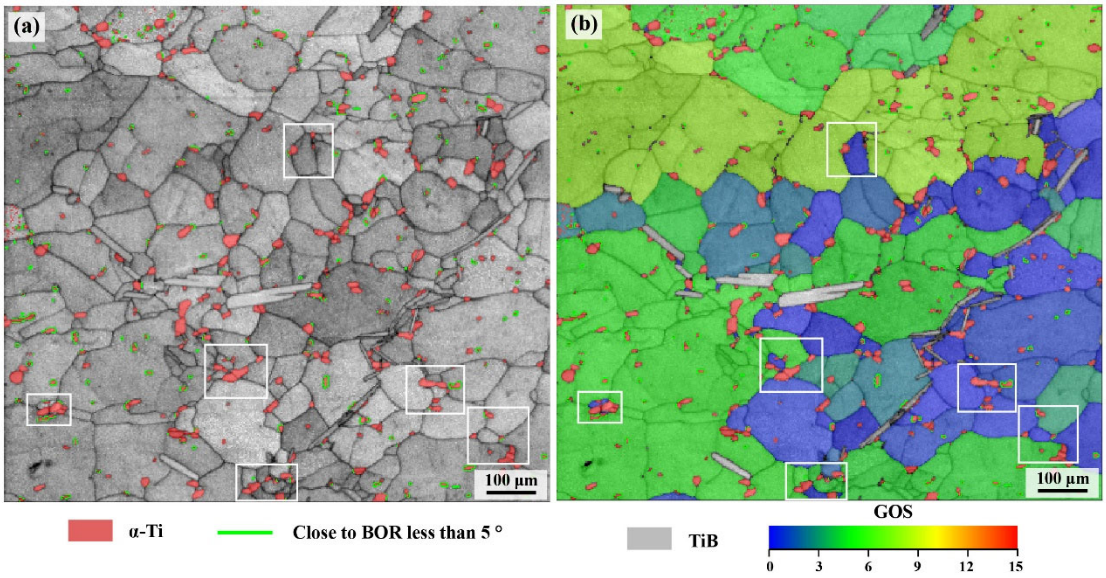

Figure S2. α-assisted DRX behavior of TiB/Ti-55531 composites compressed at 825 ℃-0.001 s^{-1} (a) BOR map, (b) GOS map
